# The Age-Related Central Auditory Processing Disorder: Silent Impairment of the Cognitive Ear

**DOI:** 10.3389/fnins.2019.00619

**Published:** 2019-06-14

**Authors:** Rodolfo Sardone, Petronilla Battista, Francesco Panza, Madia Lozupone, Chiara Griseta, Fabio Castellana, Rosa Capozzo, Maria Ruccia, Emanuela Resta, Davide Seripa, Giancarlo Logroscino, Nicola Quaranta

**Affiliations:** ^1^Unit of Epidemiological Research on Aging “Great Age Study,” National Institute of Gastroenterology-Research Hospital, IRCCS “S. De Bellis,” Bari, Italy; ^2^Istituti Clinici Scientifici Maugeri I.R.C.C.S., Institute of Cassano Murge, Bari, Italy; ^3^Geriatric Unit, Fondazione IRCCS “Casa Sollievo della Sofferenza,” Foggia, Italy; ^4^Department of Basic Medical Sciences, Neuroscience, and Sense Organs, University of Bari Aldo Moro, Bari, Italy; ^5^Department of Clinical Research in Neurology, Center for Neurodegenerative Diseases and the Aging Brain, University of Bari Aldo Moro, “Pia Fondazione Cardinale G. Panico,” Tricase, Italy; ^6^Department of Cardiac, Thoracic, and Vascular Science, Institute of Respiratory Disease, University of Bari Aldo Moro, Bari, Italy; ^7^Translational Medicine and Management of Health Systems, University of Foggia, Foggia, Italy; ^8^Otolaryngology Unit, Department of Basic Medicine, Neuroscience, and Sense Organs, University of Bari Aldo Moro, Bari, Italy

**Keywords:** age-related hearing loss, central auditory processing disorder, cognitive function, rehabilitation, dementia, MCI, lifestyle, sensorial frailty

## Abstract

Age-related hearing loss (ARHL), also called presbycusis, is a progressive disorder affecting hearing functions and among the elderly has been recognized as the third most frequent condition. Among ARHL components, the age-related central auditory processing disorder (CAPD) refers to changes in the auditory network, negatively impacting auditory perception and/or the speech communication performance. The relationship between auditory-perception and speech communication difficulties in age-related CAPD is difficult to establish, mainly because many older subjects have concomitant peripheral ARHL and age-related cognitive changes. In the last two decades, the association between cognitive impairment and ARHL has received great attention. Peripheral ARHL has recently been defined as the modifiable risk factor with the greatest impact on the development of dementia. Even if very few studies have analyzed the relationship between cognitive decline and age-related CAPD, a strong association was highlighted. Therefore, age-related CAPD could be a specific process related to neurodegeneration. Since these two disorders can be concomitant, drawing causal inferences is difficult. The assumption that ARHL, particularly age-related CAPD, may increase the risk of cognitive impairment in the elderly remains unchallenged. This review aims to summarize the evidence of associations between age-related CAPD and cognitive disorders and to define the diagnostic procedure of CAPD in the elderly. Finally, we highlight the importance of tailoring the rehabilitation strategy to this relationship. Future longitudinal studies with larger sample sizes and the use of adequate assessment tools that can disentangle cognitive dysfunction from sensory impairments are warranted.

## Introduction

Age-related hearing loss (ARHL), known as presbycusis as well, is a progressive disorder that affects hearing functions. It primarily consists of a high-frequency (4 to 8 kHz) increase of the hearing threshold (Gates and Mills, 2005). ARHL is a well-recognized condition in older age with a high prevalence in the general population, being about 20% over 65 years old but increasing to 65% over 85 years (Lin et al., 2011). Among ARHL components, the age-related central auditory processing disorder (CAPD) is defined as a peculiar deficit in the processing of auditory signals along the central auditory nervous system, including one or more areas of auditory discrimination, binaural and temporal processing, clinically featured in the elderly by the inability of understanding speech in a noisy environment (American Academy of Audiology, 2010). Two forms of CAPD are currently classified in the ICD-10 as H93.25, specifically acquired and congenital forms (World Health Organization [WHO], 2017). This disorder may be classified as developmental, acquired (i.e., as a consequence of infections, neurological diseases, stroke, or noise exposure), or secondary CAPD (British Society of Audiology [BSA], 2017). However, this classification does not include presentations like age-related CAPD, also called central presbycusis, which may affect specifically older adults (Iliadou et al., 2017). This last presentation is distinguished from the other CAPD because aging is probably the main cause. Indeed, many longitudinal and cross-sectional studies showed that the occurrence of CAPD in the elderly increases with age (Gates et al., 1996; Quaranta et al., 2014). For this reason, it has also been defined as central presbycusis (Gates, 2012).

Age-related CAPD presents specific characteristics: poor speech understanding in noisy environments, or with competing speech, or any other alteration in terms of acoustics features of speech perception (Gates, 2012). These problems can be related to degeneration of the central neural auditory pathways and are the direct consequence of the degeneration of linguistic abilities in the elderly (Rönnberg et al., 2013). However, speech perception impairment is also linked to other cognitive functions (i.e., executive and attentive functions). A clear example of this link is when listeners should match rapid acoustic input with memorized word representations and phonemes to successfully extract the proper meaning of the message. This process requires cognitive-linguistic abilities, specifically working memory (Craik, 2007; Rönnberg et al., 2013). Moreover, some longitudinal studies also suggested that age-related CAPD may be fundamental in determining an increased occurrence of incident cognitive decline and dementia such as Alzheimer’s disease (AD) (Panza et al., 2018a). This association seems to be stronger when comparing CAPD with peripheral ARHL (Yuan et al., 2018). Recently, this CAPD-cognition link has been summarized by the provocative term “the cognitive ear,” suggesting that hearing functions are not only processed by the ear and by the auditory cortex but also by other associative cortical areas (Peelle and Wingfield, 2016; Panza et al., 2018b).

The aim of the present brief review is twofold. Firstly, to summarize the evidence of associations between age-related CAPD and cognitive disorders, illustrating the usefulness of the cognitive ear construct. Secondly, to define the procedure for diagnosing CAPD in older subjects, because CAPD can be masked by the peripheral hearing deficit which is very frequent in the elderly. Indeed, the incidence of CAPD may therefore be underestimated.

## Central Auditory Functions: From the Physiology of Auditory Processing to the Clinical Diagnosis of the Disorder

Aging causes profound physiologic changes in both the peripheral and central auditory systems (Willott, 1991). The most prominent age-related hearing changes occur in the cochlea. Schuknecht and Gacek (1993) have described 4 forms of peripheral ARHL, namely sensory, neural, strial, and conductive that lead to hearing loss, and poor speech understanding. The cochlear changes responsible for peripheral ARHL have a causal role in reducing gray matter volume in the auditory cortex (Eckert et al., 2012). In particular, Lin et al. (2014) showed that ARHL is associated with shrinking of the total brain volume and, specifically, of the right temporal lobe volume. The functional consequences of ARHL are related to speech understanding which becomes more difficult (Fitzgibbons and Gordon-Salant, 2010; Humes and Dubno, 2010). Using functional MRI, Peelle and Wingfield (2016), have demonstrated that poor hearing may cause reduced language processing in primary auditory pathways and an increased compensatory language activity in other neocortical areas, not usually involved in this process, such as the prefrontal areas, the premotor cortex, and the cingulo-opercular network. This extended network, supporting language processing in presbycusis patients, has been found also by other authors, who attributed particular relevance to the cingulo-opercular cortex, involved not only in speech comprehension in normal hearing subjects but also in patients presenting with mild hearing loss (i.e., Campbell and Sharma, 2013; Sharma and Glick, 2016). Recently, the cingulo-opercular cortex has been found atrophied in a group of patients with presbycusis who also had episodic memory dysfunctions (Belkhiria et al., 2019) suggesting that cochlear dysfunction is related to cortical damage and episodic memory impairments could reflect an impairment of the Papez circuit in presbycusis patients. Overall, all these studies demonstrate that ARHL is associated with an alteration of central auditory pathways. Interestingly, the cortical areas associated with ARHL are similar to those involved in subjects with mild cognitive impairment (MCI). Thus far, cingulate cortex hypoperfusion has been found in subjects with a high risk of conversion to AD dementia (Huang et al., 2002). Several cognitive functions are processed by the cingulate cortex, one of the most important being episodic memory, which has been found as a specific neuropsychological marker in subjects with early AD (i.e., Gainotti et al., 2014 Battista et al., 2017).

Concerning CAPD, two hypotheses have been proposed to explain the origin of the disorder: the mechanistic and the neurodegeneration models (Lindenberger et al., 2001; Panza et al., 2018a). CAPD is generally defined as a peculiar impairment in the processing and analysis of auditory signals along the central auditory nervous system, referring also to the bottom-up and top-down neural connectivity (American Academy of Audiology, 2010). The former hypothesis suggests that CAPD can be the consequence of sensorial deprivation due to peripheral/cochlear damage (bottom-up theory). The neural pathways activity declines and connections are lost while the sensorial deprivation continues (Panza et al., 2018b). The other theory takes into account the strong association between CAPD and cognitive impairment, assuming the origin of CAPD to be an independent form of neurodegeneration (Humes et al., 2012). Regardless of what the correct theory may be, the causal pattern behind the alte Panza et al. (2018a) ration of central auditory pathways is still unknown (Jayakody et al., 2018). A new anatomical resource could be used, in the near future, to solve this dilemma: the human connectome, which has been developed from functional neuroimaging of thousands of healthy persons and provides a map of these brain connections. With the use of the connectome, lesions in different locations (e.g., cochlear nuclei and temporal cortex), that originated at different times, can be linked to common networks in a way that was not previously possible to identify, adding new variables to define causal inferences about the onset of age-related CAPD in normal hearing subjects (Fox, 2018).

### Cortical Auditory Functions in Aging and Cognitive Effort in Understanding Degraded Speech

At a neural level, hearing impairment leads to a reduced activation of central auditory pathways, resulting in a compensatory increased activation of the cognitive control network, as well as dysfunctional auditory–limbic connectivity, and deafferentation-induced reduction in volume of the frontal brain regions. These pathologic changes decrease cognitive performance and increase the risk of depression by reducing cognitive reserve, increasing executive dysfunction, and disrupting normative emotion reactivity and regulation (Rutherford et al., 2018).

Since the term age-related CAPD refers to the difficulty in the processing of perception of auditory information in the central nervous system, it is a neurobiological activity which underlies that processing and gives rise to the electrophysiological auditory potentials (American Speech-Language-Hearing Association, 2005). This definition matches the typical cognitive, behavioral and electrophysiological auditory outcomes associated with the aging brain.

The main cognitive tasks processed by the prefrontal cortex are executive functions, and working memory abilities seem to be specifically involved when listeners match the rapid incoming sound to the memorized representations of words and phonemes in order to successfully extract the intended meaning (Rönnberg et al., 2013). Therefore, extracting information from a degraded signal requires a greater effort, contributing to increase the cognitive effort and hence interfering with other cognitive-linguistic operations. An intuitive way to understand the role of working memory is that if an incoming signal cannot be understood, it must be maintained for a longer time to allow other cognitive systems to process it in the time it takes to function (Peelle, 2018).

### Clinical Assessment of Age-Related Central Auditory Processing Disorder

**FIGURE 1:**
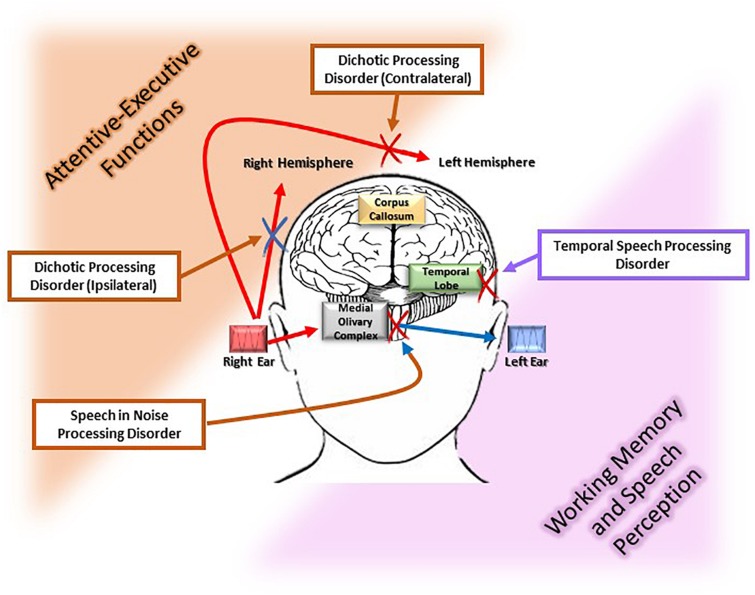
Different pathophysiological pathways of the age-related central auditory processing disorder (CAPD). The figure illustrates the myriad deficits [i.e., dichotic processing (ipsilateral and contralateral), speech recognition in noise, and temporal speech processing involved in age-related CAPD]. Each of these disorders has a particular location along the auditory pathways, i.e., superior olivary complex and corpus callosum, medial olivary complex, and temporal lobe, respectively. Moreover, each of these auditory functions is associated with a cognitive process that is susceptible to the neurodegeneration. Specifically, executive and attentive functions are associated with speech in noise processing, dichotic ipsi and contralateral processing, which are highlighted in orange in the figure, while working memory and speech perception are associated with temporal speech processing which is displayed in pink in the figure.

The most consistent clinical approach to detect age-related CAPD is by auditory behavioral assessment. At present, audiological tests have been validated primarily in subjects with a specific and well-determined impairment in the area associated with a particular auditory function (e.g., brainstem or temporal lobe tumors) (Bocca et al., 1954, 1955). In view of the many tests used in audiological practice for the definition of age-related CAPD, we have considered only those most commonly used in epidemiological studies to measure the association with cognitive impairment, as shown in Figure 1.

### Speech in Noise Processing

Understanding words in background noise becomes more challenging with the passing of years, and the elderly have significantly more difficulties with respect to younger adults (Pronk et al., 2013). Auditory processing and cognition play an important role in the intelligibility of speech in noisy environments in older age. The effect of aging is evident in understanding words in competing situations (Gates et al., 2008), time-compressed speech (Vaughan et al., 2008), and binaural speech perception (Golding et al., 2006). Anatomical pathways involved in distinguishing useful signal from noise lie in the medial olivary complex. The modulation of the medial olivary complex on the outer hair cells, to reduce the gain of noisy signals, is primarily activated in the area of attentive-executive functions (dorsolateral prefrontal cortex) (Della Penna et al., 2007).

In an observational study of about 5000 subjects aged between 40 and 60 years a drop-in speech perception against noise was observed in both sexes as from 50 years old (Moore et al., 2014). This decline was higher in subjects with lower cognitive abilities (processing speed, memory, and reasoning).

### Dichotic Processing

Dichotic listening can be defined as the contextual stimulation of both ears, with different signals reaching each ear. The role of the corpus callosum in dichotic processing has been exploited by several authors in the literature. For example, patients which present the language function lateralized in the left hemisphere, and with a clinical history of split-brain surgery, despite preoperatively normal dichotic processing abilities, presented a total inability to perform dichotic tests on left ear (Musiek et al., 1989). Jerger and Martin (2006) studied 172 older participants which present, at the Dichotic Sentence Identification test, for 58% of them, an impaired performance for the divided attention task (response required for both ears), while no alterations were found for the directed attention (response required for one ear while ignoring the other). The authors concluded that the pattern could underlying a cognitive in nature in 58% of cases and could be suggestive of structural abnormalities in the central nervous system for the 23% of cases.

A widely standardized dichotic test is by Synthetic Sentence Identification with Ipsilateral Competing Message (SSI-ICM) or a Contralateral Competing Message (SSI-CCM) (Jerger, 1973). The patient listens to an average narrative and is asked to identify 10 sentences superimposed on the narrative by the same speaker. Each sentence has no meaning even though each series of three words makes syntactical sense. The signal and the message are presented at the same intensity level, usually 40 dB above the hearing threshold. Since audibility is not an issue, the test requires the listener to attend to the message and ignore the narrative.

### Temporal Processing

Temporal processing refers to the hearing ability to individuate short changes in stimulus duration (Boettcher, 2002). Detecting and discriminating timing differences in speech is important in competitive listening or noisy environments (Schneider, 1997). Detecting gaps in continuous signals (noise or sound) is the main feature of these processes and many tests have been proposed to assess it (Phillips et al., 2000; John et al., 2012). However, temporal processes are heavily influenced by the hearing threshold and cognitive abilities such as working memory and attentive-executive functions, making it difficult to draw meaningful inferences about the effect of aging and cognitive impairment on them (Humes and Dubno, 2010). Cognitive functions such as working memory and attention decline with age, and this has a significant consequence on words understanding regardless of hearing status (Schneider et al., 2010).

### Electrophysiological Assessment

Electrophysiological measures are widely used for the evaluation of the central auditory deficits. Most studies applied highly standardized electrophysiological evaluation methods. These are subdivided topographically according to the most probable location of the bioelectric signal derivation. Auditory Brainstem Response (ABR) is an evoked potential, which is primarily free of higher-order influences via the effect of quiet, conscious rest or sleep with closed eyes. Several age-related changes of ABR have been described (Boettcher, 2002). There is little evidence regarding the influence of temporal functions on the ABR. Temporal processing can be defined as the ability to resolve rapid changes in stimulus duration and a few studies have demonstrated, introducing gap in noise stimuli, that ABRs are modified in terms of latency and amplitude (Poth et al., 2001; Goll et al., 2011). In addition to the pathological changes in auditory pathways, mouse models and AD patients also showed increased ABR thresholds, and that a greater hearing loss was related to higher adjusted relative odds for dementia (Uhlmann et al., 1989; O’Leary et al., 2017). Cortical Event-Related Potentials include a wide range of electrophysiological tests aimed at the definition of several electrical patterns of age-related auditory impairment (Jerger and Lew, 2004). The P300 seems to be a sensitive electrophysiological test for CAPD, that is thought to reflect the speed of processing of auditory information. The P300 in clinical studies often use 1,000 and 2,000 Hz tone bursts as frequent and target (oddball) stimuli, respectively, although speech signals can also be used (Polich, 2004). The comparison of P300 in terms of latency of presentation was age-dependent, while insufficient evidence was found about electrophysiological features in the link between central auditory processing and cognition (Cintra et al., 2015).

### Diagnosis of Age-Related Central Auditory Processing Disorder

There is currently no reference standard for diagnosing age-related CAPD (Vermiglio, 2016). The diagnosis is met when an alteration of the central auditory processes related to the effect of age occurs and no other cerebral pathologies can explain those alterations (Humes et al., 2012). The diagnosis of CAPD can be made if at least one of the above-described central auditory tests results impaired, as established by the corresponding cut-off scores and procedures (Humes et al., 2012).

According to Gates (2012), one of the most sensitive and widely used diagnostic tests to define age-related CAPD is the SSI test by Jerger (1973). The diagnosis made by this test is based on three criteria. Firstly, an unimpaired peripheral auditory function (<40 dB HL in the better ear) and a word recognition score in the quiet of 70% or better. Secondly, the patient must have adequate visual acuity to see the list of sentences (used to test for CAPD). Thirdly, the procedures are first administered in a “training” mode with the signal 10 dB louder than the message to ensure the patient understands the method. After that, the actual test is done at a 0-dB message-to-competition ratio; correct selection of 8 of the 10 presentations is considered normal (Gates, 2012). Moreover, the SSI-ICM appears to be more sensitive to detect dementia than the contralateral form (SSI-CCM) (Gates et al., 1995). In our clinical and research experience, we have found that the use of SSI-ICM seems to be a good standard for the diagnosis of CAPD in terms of accuracy and efficiency, and for the prediction of cognitive impairment in the elderly (Quaranta et al., 2014; Sardone et al., 2018).

In the elderly, peripheral ARHL is very common, but older individuals with impaired peripheral auditory function (>40 dB HL of pure tone average in the better ear) are not diagnosed with age-related CAPD. For this reason, nowadays the age-related CAPD component cannot be evaluated in older individuals with impaired peripheral auditory function. Possibly, the only way to solve this issue is with the use of advanced diagnostic methods that are able to bypass the cochlear input (i.e., neuroimaging methods). Consequently, the comorbidity between CAPD and peripheral ARHL could lead to underestimating the burden of the phenomenon and its consequences (Gates, 2012).

## Association Between Age-Related Central Auditory Processing Disorder and Cognitive Impairment

In the past 2 years, in cohorts studies an increasing body of meta-analytic evidence has demonstrated an increased risk of cognitive decline in peripheral ARHL (Panza et al., 2018a). Concerning age-related CAPD, few studies have investigated the relationship between this component of ARHL and cognitive impairment (Yuan et al., 2018). Specifically, this association has been found in some small-scale clinically based studies (Kurylo et al., 1993; Idrizbegovic et al., 2011, 2013; Edwards et al., 2017), two large-scale observational studies (Gates et al., 2010, 2011), one cross-sectional (Quaranta et al., 2014) and one longitudinal population-based study (Gates et al., 2002). Moreover, in the population-based Framingham cohort, Gates et al. (1996) observed that the risk of developing AD in age-related CAPD was 6.07 higher than in subjects of the same age with normal hearing. Another Italian population-based study, the Great Age Study, found an odds ratio of 11.2 in a cross-sectional survey, using the SSI-ICM test (Sardone et al., 2018).

The central component of ARHL could be the consequence of a peripheral auditory deficit, but on the other hand, central changes may be independent from peripheral ones, may be a combination of both auditory components, or, finally, a result of cognitive dysfunction. For this reason, the association between age-related CAPD and cognitive decline is usually considered only in subjects with normal hearing (Humes et al., 2012). So far, the most plausible hypothesis is that age-related CAPD and cognitive decline, particularly of executive functions, are associated (Craik, 2007).

Another way to define a pathophysiological association between age-related CAPD and cognitive decline could be to focus on the prodromal stages of dementia defined as MCI. The link between cognition and central auditory process has been demonstrated by the association between a diagnosis of MCI due to AD and poorer performance on tests of central auditory processing (i.e., Idrizbegovic et al., 2011). Furthermore, Iliadou and Kaprinis (2003) reviewed the literature and concluded that CAPD may be a precursor of AD, preceding the clinical diagnosis by 5 to 10 years. However, even if most of these clinical and epidemiological studies suggest a link between central auditory dysfunction and cognitive decline, the causal mechanisms underlying this link are still unknown (Wayne and Johnsrude, 2015). Moreover, although an association has been observed, no consistent data with longitudinal evidence are still available (Idrizbegovic et al., 2011, 2013; Quaranta et al., 2014; Edwards et al., 2017).

A seminal neuropathological study supported the hypothesis that age-related CAPD may result from a degenerative pathway other than cognitive decline, showing that brain amyloid-βββ, believed to be the initial event of AD, was uncommon in central auditory pathways early in the clinical course of the disease. By contrast, there was early formation of neurofibrillary tangles, mainly consisting of hyperphosphorylated tau protein, suggesting that neurodegeneration in the auditory system may be an ongoing process throughout the AD course (Sinha et al., 1993).

Therefore, further research is needed in order to disentangle the real causal association between pathological correlates of presbycusis (including cochlear receptor cell loss, stria vascularis atrophy, and auditory-nerve neuron loss) and the atrophy of specific brain regions, and consequently the related cognitive domains involved in subjects with CAPD (Shen et al., 2018). The only way to observe whether the neurodegenerative process starts from the frontal lobe (executive functions) or from the central auditory pathways could be through further longitudinal studies on a generalizable population with dynamic neuroimaging features.

## Clinical Implications and Rehabilitation

It is very difficult to devise rehabilitation strategies for a silent deficit like age-related CAPD, especially if associated with an initial cognitive impairment. One of the most logic approaches is to increase the listener’s signal/noise ratio, controlling the acoustic environment in order to decrease listening difficulties. Reducing noise and reverberation and increasing the direct sound field (lower ceilings, less reverberating materials, and more preferential seating). This is also a top-down strategy aimed at reducing the cognitive load (Baran, 2002). Another strategy, this time bottom-up, in subjects with hearing aids could be to increase the gain of the near field signal, using a frequency-modulation system: listening skill may be further enhanced by frequency-modulation systems (Kricos, 2006) and using binaural stimulation in the hearing aids (increasing the loudness summation and localization of the sound source) (Walden and Walden, 2005).

Since age related central auditory dysfunction may be involved in the continuum from preclinical to advanced stages of dementia (Jayakody et al., 2018), it is very important to implement holistic and structured intervention for these subjects. Specifically, this intervention should combine auditory and cognitive functions, increasing sensorial input and decreasing the cognitive effort/working memory ability, respectively (see Figure 1).

**FIGURE 2:**
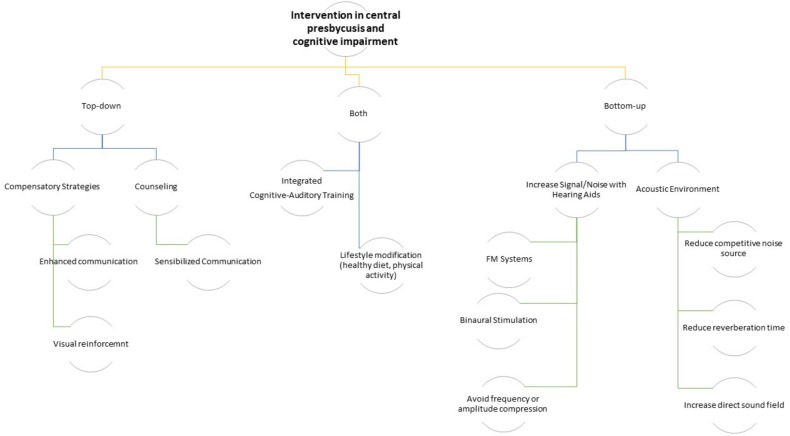
Logical algorithm tree of possible interventions on the age-related CAPD in cognitively impaired older subjects.

Caregivers together with patients who are experiencing contextually age-related CAPD and cognitive impairment should undergo counseling with audiologists and neuropsychologists to start awareness training that includes instruction in the use of meaningful gestures and beneficial conversational techniques such as speaking more clearly, with long intervals, and with more enunciation (Kiessling et al., 2003). A rehabilitation protocol that includes both hearing training and a cognitive rehabilitation may be useful to delay the synergic effect of both deficits. Additionally, individuals with age-related CAPD may improve the subjective impairment participating in individual and/or group training sessions (Figure 2; Heine and Browning, 2002). Therefore, it is evident that intervention on all risk factors that cause both cognitive impairment and hearing loss may, in some way, slow down the vicious circle of reverse causality, i.e., intervening on lifestyle factors. A diet fulfilling the Mediterranean-type dietary pattern with a high presence of antioxidant-rich foods and an anaerobic physical activity for at least 20 minutes 5 days a week could be recommended as a useful adjuvant to the combined rehabilitation therapy for hearing loss and cognitive impairment (Figure 2; Solfrizzi et al., 2018). However, the evidence supporting the effectiveness of any particular intervention approach is relatively weak due to the lack of good quality interventional studies.

## Future Directions and Conclusion

The central auditory deficit is a silent impairment because it does not have an immediate impact on daily functions. As consequences, two fundamental problems arise. The first is that the subject is usually not aware of his/her deficits and tends to minimize the handicap, often avoiding situations that could trigger it, such as avoiding noisy or crowded places. This predisposes patients to a social isolation that has been shown to have an important effect on cognitive status (Lozupone et al., 2018). The second point is that age-related CAPD is often associated with a deficit of some cognitive domains, in particular executive functions. This could mask the coexistence of the two conditions and delay the institution of rehabilitative and preventive interventions.

When the clinical suspicion of age-related CAPD and cognitive impairment is confirmed, clinicians should be sufficiently aware to be able to address patients to rehabilitation programs. The first target of future research should be to improve the diagnosis of CAPD in the elderly, when cognitive disorders are present, relying on the identification of specific psychoacoustic, clinical, or objective auditory markers (core measures) to guide focused management provision. Finally, fundamental pathways to radically change the trajectories of the degeneration of the “cognitive ear” lie in two future directions. The first direction will involve high quality audiological assessment supported by a targeted use of next generation neuroimaging. The second direction lies in demonstrating the benefits of next generation hearing restoration devices (cochlear implants and hearing aids) empowered with artificial intelligence algorithms in order to reduce competitive acoustic signals. The rehabilitative outcomes of these devices need to be tested by means of well-designed controlled clinical trials, during tailored intervention on cognitive impairment and CAPD.” (Jayakody et al., 2018; Panza et al., 2018a).

## Author Contributions

RS, PB, FP, GL, and NQ contributed substantially to the conception and design of the work, drafting and revising the manuscript for important intellectual content, approved the final version to be published, and agreed to be accountable for all aspects of the work. CG, FC, RC, ML, MR, ER, and DS drafted corresponding sections of the manuscript. All authors approved the final version to be published and agreed to be accountable for all aspects of the work.

## Conflict of Interest Statement

The authors declare that the research was conducted in the absence of any commercial or financial relationships that could be construed as a potential conflict of interest.

## References

[B1] American Academy of Audiology (2010). *Diagnosis, Treatment and Management of Children and Adults with Central Auditory Processing Disorder.* Available at: https://audiology-web.s3.amazonaws.com/migrated/CAPD%20Guidelines%208-2010.pdf_539952af956c79.73897613.pdf (accessed May 31, 2019).

[B2] American Speech-Language-Hearing Association (2005). *(Central) Auditory Processing Disorders [Technical Report].* Available at: http://www.asha.org/policy/TR2005-00043/ (accessed May 31, 2019).

[B3] Baran J. (2002). Managing auditory processing disorders in adolescents and adults. *Semin. Hear.* 23 327–336. 10.1055/s-2002-35881

[B4] Battista P. Salvatore C. Castiglioni I. (2017). Optimizing neuropsychological assessments for cognitive, behavioral, and functional impairment classification: a machine learning study. *Behav. Neurol.* 2017:1850909. 10.1155/2017/1850909 28255200 PMC5307249

[B5] Belkhiria C. Vergara R. C. San Martin S. Leiva A. Marcenaro B. Martinez M. (2019). Cingulate cortex atrophy is associated with hearing loss in presbycusis with cochlear amplifier dysfunction. *Front. Aging Neurosci.* 11:97. 10.3389/fnagi.2019.00097 31080411 PMC6497796

[B6] Bocca E. Calearo C. Cassinari V. (1954). A new method for testing hearing in temporal lobe tumours; preliminary report. *Acta Otolaryngol.* 44 219–221. 10.3109/00016485409128700 13197002

[B7] Bocca E. Calearo C. Cassinari V. Migliavacca F. (1955). Testing “cortical” hearing in temporal lobe tumours. *Acta Otolaryngol.* 45 289–304. 10.3109/00016485509124282 13275293

[B8] Boettcher F. A. (2002). Presbycusis and the auditory brainstem response. *J. Speech Lang. Hear. Res.* 45 1249–1261. 12546491 10.1044/1092-4388(2002/100)

[B9] British Society of Audiology [BSA] (2017). *Position Statement and Practice Guidance, Auditory Processing Disorder (APD).* Available at: http://www.thebsa.org.uk/wp-content/uploads/2017/04/APD-Position-Statement-Practice-Guidance-APD-2017.pdf (accessed May 31, 2019).

[B10] Campbell J. Sharma A. (2013). Compensatory changes in cortical resource allocation in adults with hearing loss. *Front. Syst. Neurosci.* 7:71 10.3389/fnsys.2013.00071 PMC3905471 24478637

[B11] Cintra M. T. G. Tavares M. S. Gomes S. A. de Oliveira Gonçalves T. Matos da Cunha L. C. Utsch Gonçalves D. (2015). P300 evoked potential and risk of mild cognitive impairment progression to Alzheimer’s dementia: a literature review. *J. Neurol. Neurophysiol.* 6:322.

[B12] Craik F. I. (2007). The role of cognition in age-related hearing loss. *J. Am. Acad. Audiol.* 18 539–547. 10.3766/jaaa.18.7.2 18236642

[B13] Della Penna S. Brancucci A. Babiloni C. Franciotti R. Pizzella V. Rossi D. (2007). Lateralization of dichotic speech stimuli is based on specific auditory pathway interactions. *Cereb. Cortex* 17 2303–2311. 10.1093/cercor/bhl139 17170048

[B14] Eckert M. A. Cute S. L. Vaden K. I. Kuchinsky S. E. Dubno J. R. (2012). Auditory cortex signs of age-related hearing loss. *J. Assoc. Res. Otolaryngol.* 13 703–713. 10.1007/s10162-012-0332-5 22618352 PMC3441956

[B15] Edwards J. D. Lister J. J. Elias M. N. Tetlow A. M. Sardina A. L. Sadeq N. A. (2017). Auditory processing of older adults with probable mild cognitive impairment. *J. Speech Lang. Hear. Res.* 60 1427–1435. 10.1044/2016_JSLHR-H-16-0066 28510618

[B16] Fitzgibbons P. J. Gordon-Salant S. (2010). *Behavioral Studies with Aging Humans: Hearing Sensitivity and Psychoacoustics.* New York, NY: Springer, 111–134.

[B17] Fox M. D. (2018). Mapping symptoms to brain networks with the human connectome. *N. Engl. J. Med.* 379 2237–2245. 10.1056/nejmra1706158 30575457

[B18] Gainotti G. Quaranta D. Vita M. G. Marra C. (2014). Neuropsychological predictors of conversion from mild cognitive impairment to Alzheimer’s disease. *J. Alzheimers Dis.* 38 481–495. 10.3233/jad-130881 24002185

[B19] Gates G. A. (2012). Central presbycusis: an emerging view. *Otolaryngol. Head Neck Surg.* 147 1–2. 10.1177/0194599812446282 22535914

[B20] Gates G. A. Anderson M. L. McCurry S. M. Feeney M. P. Larson E. B. (2011). Central auditory dysfunction as a harbinger of Alzheimer dementia. *Arch. Otolaryngol. Head Neck Surg.* 137 390–395. 10.1001/archoto.2011.28 21502479 PMC3170925

[B21] Gates G. A. Beiser A. Rees T. S. D’Agostino R. B. Wolf P. A. (2002). Central auditory dysfunction may precede the onset of clinical dementia in people with probable Alzheimer’s disease. *J. Am. Geriatr. Soc.* 50 482–488. 10.1046/j.1532-5415.2002.50114.x 11943044

[B22] Gates G. A. Cobb J. L. Linn R. T. Rees T. Wolf P. A. D’Agostino R. B. (1996). Central auditory dysfunction, cognitive dysfunction, and dementia in older people. *Arch. Otolaryngol. Head Neck Surg.* 122 161–167. 10.1001/archotol.1996.01890140047010 8630210

[B23] Gates G. A. Feeney M. P. Mills D. (2008). Cross-sectional age-changes of hearing in the elderly. *Ear Hear.* 29 865–874. 10.1097/aud.0b013e318181adb5 18998241

[B24] Gates G. A. Gibbons L. E. McCurry S. M. Crane P. K. Feeney M. P. Larson E. B. (2010). Executive dysfunction and presbycusis in older persons with and without memory loss and dementia. *Cogn. Behav. Neurol.* 23 218–223. 10.1097/WNN.0b013e3181d748d7 21150347 PMC3058598

[B25] Gates G. A. Karzon R. K. Garcia P. Peterein J. Storandt M. Morris J. C. (1995). Auditory dysfunction in aging and senile dementia of the Alzheimer’s type. *Arch. Neurol.* 52 626–634. 7763213 10.1001/archneur.1995.00540300108020

[B26] Gates G. A. Mills J. H. (2005). Presbycusis. *Lancet* 366 1111–1120. 16182900 10.1016/S0140-6736(05)67423-5

[B27] Golding M. Taylor A. Cupples L. Mitchell P. (2006). Odds of demonstrating auditory processing abnormality in the average older adult: the blue mountains hearing study. *Ear Hear.* 27 129–138. 10.1097/01.aud.0000202328.19037.ff 16518141

[B28] Goll J. C. Kim L. G. Hailstone J. C. Lehmann M. Buckley A. Crutch S. J. (2011). Auditory object cognition in dementia. *Neuropsychologia* 49 2755–2765. 10.1016/j.neuropsychologia.2011.06.004 21689671 PMC3202629

[B29] Heine C. Browning C. J. (2002). Communication and psychosocial consequences of sensory loss in older adults: overview and rehabilitation directions. *Disabil. Rehabil.* 24 763–773. 10.1080/09638280210129162 12437862

[B30] Huang C. Wahlund L. O. Svensson L. Winblad B. Julin P. (2002). Cingulate cortex hypoperfusion predicts Alzheimer’s disease in mild cognitive impairment. *BMC Neurol.* 2:9. 10.1186/1471-2377-2-9 12227833 PMC128832

[B31] Humes L. E. Dubno J. R. (2010). *Factors Affecting Speech Understanding in Older Adults.* New York, NY: Springer, 211–257.

[B32] Humes L. E. Dubno J. R. Gordon-Salant S. Lister J. J. Cacace A. T. Cruickshanks K. J. (2012). Central presbycusis: a review and evaluation of the evidence. *J. Am. Acad. Audiol.* 23 635–666. 10.3766/jaaa.23.8.5 22967738 PMC5898229

[B33] Idrizbegovic E. Hederstierna C. Dahlquist M. Nordström C. K. Jelic V. Rosenhall U. (2011). Central auditory function in early Alzheimer’s disease and in mild cognitive impairment. *Age Ageing* 40 249–254. 10.1093/ageing/afq168 21233090

[B34] Idrizbegovic E. Hederstierna C. Dahlquist M. Rosenhall U. (2013). Short-term longitudinal study of central auditory function in Alzheimer’s disease and mild cognitive impairment. *Dement. Geriatr. Cogn. Dis. Extra* 3 468–471. 10.1159/000355371 24516414 PMC3919463

[B35] Iliadou V. Kaprinis S. (2003). Clinical psychoacoustics in Alzheimer’s disease central auditory processing disorders and speech deterioration. *Ann. Gen. Hosp. Psychiatry* 2:12. 14690547 10.1186/1475-2832-2-12 PMC317473

[B36] Iliadou V. V. Ptok M. Grech H. Pedersen E. R. Brechmann A. Deggouj N. (2017). A european perspective on auditory processing disorder-current knowledge and future research focus. *Front. Neurol.* 8:622. 10.3389/fneur.2017.00622 29209272 PMC5702335

[B37] Jayakody D. M. Friedland P. L. Martins R. N. Sohrabi H. R. (2018). Impact of aging on the auditory system and related cognitive functions: a narrative review. *Front. Neurosci.* 12:125. 10.3389/fnins.2018.00125 29556173 PMC5844959

[B38] Jerger J. (1973). Audiological findings in aging. *Adv. Otorhinolaryngol.* 20 115–124. 4710504

[B39] Jerger J. Lew H. L. (2004). Principles and clinical applications of auditory evoked potentials in the geriatric population. *Phys. Med. Rehabil. Clin. N. Am*. 151 235–250. 10.1016/s1047-9651(03)00099-8 15029907

[B40] Jerger J. Martin J. (2006). Dichotic listening tests in the audiological assessment of auditory processing disorders. *Audiol. Med.* 4 25–34. 10.1080/16513860600567823

[B41] John A. B. Hall J. W. Kreisman B. M. (2012). Effects of advancing age and hearing loss on gaps-in-noise test performance. *Am. J. Audiol.* 21 242–250. 10.1044/1059-0889(2012/11-0023) 22992446

[B42] Kiessling J. Pichora-Fuller M. K. Gatehouse S. Stephens D. Arlinger S. Chisolm T. (2003). Candidature for and delivery of audiological services: special needs of older people. *Int. J. Audiol.* 42(Suppl. 2), S92–S101. 12918635

[B43] Kricos P. B. (2006). Audiologic management of older adults with hearing loss and compromised cognitive/psychoacoustic auditory processing capabilities. *Trends Amplif.* 10 1–28. 10.1177/108471380601000102 16528428 PMC4111542

[B44] Kurylo D. D. Corkin S. Allard T. Zatorre R. J. Growdon J. H. (1993). Auditory function in Alzheimer’s disease. *Neurology* 43 1893–1899. 8413944 10.1212/wnl.43.10.1893

[B45] Lin F. R. Ferrucci L. An Y. Goh J. O. Doshi J. Metter E. J. (2014). Association of hearing impairment with brain volume changes in older adults. *Neuroimage* 90 84–92. 10.1016/j.neuroimage.2013.12.059 24412398 PMC3951583

[B46] Lin F. R. Thorpe R. Gordon-Salant S. Ferrucci L. (2011). Hearing loss prevalence and risk factors among older adults in the United States. *J. Gerontol. A Biol. Sci. Med. Sci*. 66 582–590. 10.1093/gerona/glr002 21357188 PMC3074958

[B47] Lindenberger U. Scherer H. Baltes P. B. (2001). The strong connection between sensory and cognitive performance in old age: not due to sensory acuity reductions operating during cognitive assessment. *Psychol. Aging* 16 196–205. 10.1037/0882-7974.16.2.196 11405308

[B48] Lozupone M. Panza F. Piccininni M. Copetti M. Sardone R. Imbimbo B. P. (2018). Social dysfunction in older age and relationships with cognition, depression, and apathy: the GreatAGE study. *J. Alzheimers Dis.* 65 989–1000. 10.3233/JAD-180466 30103335

[B49] Moore D. R. Edmondson-Jones M. Dawes P. Fortnum H. McCormack A. Pierzycki R. H. (2014). Relation between speech-in-noise threshold, hearing loss and cognition from 40–69 years of age. *PLoS One* 9:e107720. 10.1371/journal.pone.0107720 25229622 PMC4168235

[B50] Musiek F. E. Kurdziel-Schwan S. Kibbe K. S. Gollegly K. M. Baran J. A. Rintelmann W. F. (1989). The dichotic rhyme task: results in split-brain patients. *Ear Hear.* 10 33–39. 10.1097/00003446-198902000-00006 2721826

[B51] O’Leary T. P. Shin S. Fertan E. Dingle R. N. Almuklass A. Gunn R. K. (2017). Reduced acoustic startle response and peripheral hearing loss in the 5xFAD mouse model of Alzheimer’s disease. *Genes Brain Behav.* 16 554–563. 10.1111/gbb.12370 28133939

[B52] Panza F. Lozupone M. Sardone R. Battista P. Piccininni M. Dibello V. (2018a). Sensorial frailty: age-related hearing loss and the risk of cognitive impairment and dementia in later life. *Ther. Adv. Chronic Dis.* 10.1177/2040622318811000 [Epub ahead of print]. PMC6700845 31452865

[B53] Panza F. Quaranta N. Logroscino G. (2018b). Sensory changes and the hearing loss–cognition link: the cognitive ear. *JAMA Otolaryngol. Head Neck Surg.* 144 127–128. 29222539 10.1001/jamaoto.2017.2514

[B54] Peelle J. E. (2018). Listening effort: how the cognitive consequences of acoustic challenge are reflected in brain and behavior. *Ear Hear.* 39 204–214. 10.1097/AUD.0000000000000494 28938250 PMC5821557

[B55] Peelle J. E. Wingfield A. (2016). The neural consequences of age-related hearing loss. *Trends Neurosci.* 39 486–497. 10.1016/j.tins.2016.05.001 27262177 PMC4930712

[B56] Phillips S. L. Gordon-Salant S. Fitzgibbons P. J. Yeni-Komshian G. (2000). Frequency and temporal resolution in elderly listeners with good and poor word recognition. *J. Speech Lang. Hear. Res.* 43 217–228. 10.1044/jslhr.4301.217 10668664

[B57] Polich J. (2004). Clinical application of the P300 event-related brain potential. *Phys. Med. Rehabil. Clin. N. Am*. 15 133–161. 10.1016/s1047-9651(03)00109-8 15029903

[B58] Poth E. A. Boettcher F. A. Mills J. H. Dubno J. R. (2001). Auditory brainstem responses in younger and older adults for broadband noises separated by a silent gap. *Hear. Res.* 161 81–86. 10.1016/s0378-5955(01)00352-5 11744284

[B59] Pronk M. Deeg D. J. Festen J. M. Twisk J. W. Smits C. Comijs H. C. (2013). Decline in older persons’ ability to recognize speech in noise: the influence of demographic, health-related, environmental, and cognitive factors. *Ear Hear.* 34 722–732. 10.1097/AUD.0b013e3182994eee 24165301

[B60] Quaranta N. Coppola F. Casulli M. Barulli M. R. Panza F. Tortelli R. (2014). The prevalence of peripheral and central hearing impairment and its relation to cognition in older adults. *Audiol. Neurootol.* 19(Suppl. 1), 10–14. 10.1159/000371597 25733360

[B61] Rönnberg J. Lunner T. Zekveld A. Sörqvist P. Danielsson H. Lyxell B. (2013). The ease of language understanding (ELU) model: theoretical, empirical, and clinical advances. *Front. Syst. Neurosci.* 7:31. 10.3389/fnsys.2013.00031 23874273 PMC3710434

[B62] Rutherford B. R. Brewster K. Golub J. S. Kim A. H. Roose S. P. (2018). Sensation and psychiatry: linking age-related hearing loss to late-life depression and cognitive decline. *Am. J. Psychiatry* 175 215–224. 10.1176/appi.ajp.2017.17040423 29202654 PMC5849471

[B63] Sardone R. Battista P. Tortelli R. Piccininni M. Coppola F. Guerra V. (2018). Relationship between central and peripheral presbycusis and mild cognitive impairment in a population-based study of Southern Italy: the “Great Age Study”. *Neurology* 90(15 Suppl.):P1.131.

[B64] Schneider B. (1997). Psychoacoustics and aging: implications for everyday listening. *J. Speech Lang. Pathol. Audiol.* 21 111–124.

[B65] Schneider B. A. Pichora-Fuller K. Daneman M. (2010). *Effects of Senescent Changes in Audition and Cognition on Spoken Language Comprehension.* New York, NY: Springer, 167–210.

[B66] Schuknecht H. F. Gacek M. R. (1993). Cochlear pathology in presbycusis. *Ann. Otol. Rhinol. Laryngol.* 102(1 Pt 2), 1–16. 10.1177/00034894931020s101 8420477

[B67] Sharma A. Glick H. (2016). Cross-modal re-organization in clinical populations with hearing loss. *Brain Sci.* 6:4. 10.3390/brainsci6010004 26821049 PMC4810174

[B68] Shen Y. Ye B. Chen P. Wang Q. Fan C. Shu Y. (2018). Cognitive decline, dementia, alzheimer’s disease and presbycusis: examination of the possible molecular mechanism. *Front. Neurosci.* 12:394 10.3389/fnins.2018.00394 PMC6002513 29937713

[B69] Sinha U. K. Hollen K. M. Rodriguez R. Miller C. A. (1993). Auditory system degeneration in Alzheimer’s disease. *Neurology* 43 779–785. 8469340 10.1212/wnl.43.4.779

[B70] Solfrizzi V. Agosti P. Lozupone M. Custodero C. Schilardi A. Valiani V. (2018). Nutritional interventions and cognitive-related outcomes in patients with late-life cognitive disorders: a systematic review. *Neurosci. Biobehav. Rev.* 95 480–498. 10.1016/j.neubiorev.2018.10.022 30395922

[B71] Uhlmann R. F. Larson E. B. Rees T. S. Koepsell T. D. Duckert L. G. (1989). Relationship of hearing impairment to dementia and cognitive dysfunction in older adults. *JAMA* 261 1916–1919. 10.1001/jama.261.13.1916 2926927

[B72] Vaughan N. Storzbach D. Furukawa I. (2008). Investigation of potential cognitive tests for use with older adults in audiology clinics. *J. Am. Acad. Audiol.* 197 533–541. 10.3766/jaaa.19.7.2 19248729

[B73] Vermiglio A. J. (2016). On diagnostic accuracy in audiology: central site of lesion and central auditory processing disorder studies. *J. Am. Acad. Audiol.* 27 141–156. 10.3766/jaaa.15079 26905533

[B74] Walden T. C. Walden B. E. (2005). Unilateral versus bilateral amplification for adults with impaired hearing. *J. Am. Acad. Audiol.* 16 574–584. 10.3766/jaaa.16.8.6 16295244

[B75] Wayne R. V. Johnsrude I. S. (2015). A review of causal mechanisms underlying the link between age-related hearing loss and cognitive decline. *Ageing Res. Rev.* 23 154–166. 10.1016/j.arr.2015.06.002 26123097

[B76] Willott J. F. (1991). *Aging and the Auditory System: Anatomy, Physiology, and Psychophysics.* San Diego, CA: Singular Publishing Group.

[B77] World Health Organization [WHO] (2017). *Deafness and Hearing Loss, Fact Sheet.* Available at: http://www.who.int/mediacentre/factsheets/fs300/en/ (accessed May 31, 2019).

[B78] Yuan J. Sun Y. Sang S. Pham J. H. Kong W. J. (2018). The risk of cognitive impairment associated with hearing function in older adults: a pooled analysis of data from eleven studies. *Sci. Rep.* 8:2137. 10.1038/s41598-018-20496-w 29391476 PMC5794920

